# The short-form of the Cyberchondria Severity Scale (CSS-12): Adaptation and validation of the Spanish version in young Peruvian students

**DOI:** 10.1371/journal.pone.0292459

**Published:** 2023-10-05

**Authors:** Rodrigo Robles-Mariños, Germán F. Alvarado, Jorge L. Maguiña, Juan Carlos Bazo-Alvarez

**Affiliations:** 1 School of Medicine, Universidad Peruana de Ciencias Aplicadas (UPC), Lima, Peru; 2 Research Department of Primary Care and Population Health, University College London (UCL), London, United Kingdom; 3 Universidad Privada Norbert Wiener, Lima, Peru; National Cheng Kung University College of Medicine, TAIWAN

## Abstract

**Background:**

Cyberchondria is defined as the increase in health-related anxiety or anguish associated with excessive or repeated online searches for health-related information. Our objective was to cross-culturally adapt and validate the CSS-12 scale for Peruvian Spanish speakers, to determine whether the Bifactor model works as well in our population as in previous studies’ and to explore whether the Bifactor-ESEM is a more suitable model.

**Methods:**

We performed a cultural adaptation using the Delphi method and a validation study on medical students between 2018 and 2019. Reliability was evaluated by using Cronbach’s alpha (α) and McDonald’s omega (Ω) for internal consistency, and Pearson’s r and intraclass correlation coefficient (ICC), for test-retest reliability. We evaluated construct validity by contrasting four measurement models for the CSS-12 and the convergent validity against health anxiety.

**Results:**

The Spanish CSS-12 showed excellent reliability (α = .93; Ω = .93; ICC = .93; r = .96). The Bifactor ESEM model showed the best fit, supporting a unidimensional measure of the general cyberchondria. This measure was positively associated with health anxiety (r = .51).

**Conclusions:**

The Spanish CSS-12 provides a valid and reliable unidimensional measure of cyberchondria, which is distinguishable from the more general health anxiety. This can be applied to similar populations and future research. The Bifactor-ESEM model appears to offer a more accurate and realistic representation of the multifaceted nature of cyberchondria. We provide a free-to-use form of the Spanish CSS-12 as supplemental material.

## Introduction

The increased distress or anxiety related to repeated or excessive searches online for health-related information is defined as “cyberchondria” (CYB) [1, 2]. CYB is considered an abnormal behaviour pattern, rather than a diagnostic entity or condition [3], and it is widespread among people with high levels of “health anxiety” (HA) [4], which is defined as a state of excessive, unrealistic and persistent worry regarding the suffering of disease [5]. In the absence of significant anxiety, searching for health information online could be a precursor to increased HA [6]; or rather, HA could be a top motivator for searching health-related information online [7]. In 2013, a representative survey in the US reported that 35% of people used the Internet to self-diagnose a medical condition [8]. In 2015, a study reported that 70% of adults used the Internet as their first source for searching health-related information [9].

High levels of HA associated with increased access to health-related information on the Internet can carry considerable repercussions. For example, it can affect work and social development since too much time is spent investigating possible diseases [10]. Furthermore, it predisposes the individual to seek medical attention, undergoes unnecessary exams, wastes medical resources, and creates exposure to possible adverse effects when self-prescription occurs [10–12]. Persons with HA can repeat this behaviour in many opportunities [10, 13] and increase the risk of suicide [14].

The Cyberchondria Severity Scale (CSS) is a self-report instrument designed to facilitate this construct’s multidimensional measure [15]. The CSS consists of 33 items distributed in 5 domains: Excessiveness (escalating/repeated nature of searches), Distress (negative emotional response), Reassurance (searching drives individuals to seek out professional medical advice), Compulsion (web searches interfering with other aspects of on/offline life) and Mistrust (conflict arising when medical professional and online self-diagnosis do not align). Created in English, the CSS has been adapted to different languages such as Turkish [16], German [17] and Portuguese (Brazil) [18]. Their psychometric properties have been reviewed in posterior studies [19–22], arguing differences regarding the more plausible measurement model. In particular, an independent study suggested a Bifactor model for the CSS (General CYB factor, which is orthogonal to its subfactors), concluding that Mistrust should be removed from the poll of domains [20].

Based on this evidence, the original authors developed a short version of the CSS -known as the Cyberchondria Severity Scale– 12 (CSS-12)–to overcome the limitations reported by others [23]. In the CSS-12, the domain Mistrust was eliminated because of its theoretical ambiguity, lack of load in the general CYB factor and low correlation with other domains [19, 20]. Besides, this version only considered three optimal items for each domain due to criticism about the extension of the original version and regarding items that could be omitted because they are not relevant or specific [3, 17]. Thus, the CCS-12 assesses CYB through 4 domains: Excessiveness, Distress, Reassurance and Compulsion, a structure validated through a Bifactor model [23].

Although the Bifactor model has demonstrated adequate psychometric properties, testing a Bifactor-ESEM model is relevant given the intriguing patterns within the cyberchondria [15]. For example, certain Excessiveness items have exhibited a tendency to shift into the Distress and Reassurance dimensions [17]. Items such as Item 5, which did not load within its designated dimension during the original CSS-12 validation [23], raise questions about a potential shared loading with other CSS-12 dimensions.

The CSS-12 is a more robust version to evaluate CYB, but it is not available in Spanish. Indeed, there are no instruments for assessing CYB in Spanish-speakers, partially explaining the absence of research about this critical topic in Latin America. Our objective was to cross-culturally adapt and validate the CSS-12 scale for Peruvian Spanish speakers. We also wanted to determine whether the Bifactor model works as well in our population as in previous studies’ and to explore whether the Bifactor-ESEM is a more suitable model.

## Methods

We developed this study in two steps: (a) adaptation to Spanish and (b) psychometric validation. The latter included evaluating the adapted CSS-12 regarding reliability, structure validity and external validity against an independent measure of anxiety.

### (a) Adaptation to Spanish

The original version of the CSS-12 [23] was independently translated into Spanish by two native Peruvians proficient in English for performing a cultural adaptation. Both translations were back-translated by two native English speakers who are proficient in Spanish [24]. The two final translations in English were then compared with the original CSS-12, and we selected the translated items most like the original ones. After that, the Spanish counterparts of the selected items were put together, and a harmonization process was carried out with five experts, using the Escobar-Pérez’s instrument for the Delphi method [25]. Five categories were evaluated with this instrument: sufficiency, clarity, coherence, relevance and cultural adaptation. Each of the 12 items was evaluated, considering the five categories and using a Likert scale from 1 to 4. The Delphi method finished when all the experts scored ≥3 in each of the 12 items finally delivered.

### (b) Psychometric validation

#### Participants

We performed a psychometric validation of the adapted CSS-12 on medical students recruited from a Peruvian university. We selected this population because the original CSS-12 was validated in college students; thus, it facilitates comparisons between both studies. We planned to evaluate at least 360 participants (i.e., 30 evaluations * 12 items = 360 evaluations), following standard recommendations for the statistical analysis described below [26]. We initially selected 764 participants from a census of all students enrolled between the second and sixth cycles of the medical career; however, we virtually surveyed 674 students (S1 Fig). Then, 17 were eliminated due to refusing informed consent or an incomplete completion of the survey. Finally, 657 surveys were analyzed, from which 396 (60.3%) were women. The average age of overall participants was 21.3 years (standard deviation (SD) = 2.2). Most of the sample was born in Lima (64%), the Peruvian capital, followed by Peruvian provinces (33%) and other countries (3%). The sample was primarily from the fourth year (29%), followed by second (26%), third (20%), sixth (14%), and fifth year (11%).

#### Reliability

We evaluated the reliability by assessing the internal consistency of the CSS-12 items using the 657 cross-sectional applications. Cronbach’s alpha and McDonald’s omega were calculated to assess the general scale’s internal consistency and per each domain [27, 28]. Additionally, we performed a test-retest evaluation in 30 extra participants with similar characteristics to the primary sample (N = 657), who agreed to participate freely and gave their consent. For this evaluation, the instrument was applied twice -seven days apart [29] to assess how stable over time the CSS-12 is, considering the general scale and each domain. We calculated both the Pearson’s correlation coefficient (Pearson’s r) and intraclass correlation coefficient (ICC) for this analysis.

#### Validity

We evaluated the construct validity of the CSS-12 and its convergent validity against an indicator of HA. For the latter, we applied the Spanish version of the Short Health Anxiety Inventory (SHAI) [30, 31]. We calculated the correlation (Pearson’s r) between total scores of SHAI and CSS-12, proceeding similarly with each of its dimensions. For evaluating the construct validity, we contrasted the one-factor, four-factor and Bifactor models of the CSS-12 as McElroy et al. did previously [23] but applying the Exploratory Structural Equation Modelling (ESEM) approach [32]. This approach allows the items to load on all dimensions (exploratory component), giving more load to the domain we know is more explicative of the item (confirmatory component). This gives access to all the usual SEM parameters, and the loading rotation also gives a transformation of the structural coefficients [33]. Since some of the CSS-12 items can be explained by more than one dimension [17, 20], the ESEM approach is more appropriate for evaluating the internal structure of the CSS-12. Details of the statistical modelling and comparison will be provided later in this section.

##### Cyberchondria Severity Scale-12 (CSS-12)

The CSS-12 [23] uses a Likert-type scale from 1 (never) to 5 (always). Four domains of CYB are measured by three items each: Excessiveness (EXC), Distress (DIST), Reassurance (REAS), and Compulsion (COMP) (all described in the introduction). As we explained above, the CSS-12 does not include the Mistrust domain due to its inconsistency with the other domains. The overall measure goes from 12 to 60 (between 3 and 15 each domain). In college students from the UK, the original version of the CSS-12 [23] showed high reliability for the general scale (α = 0.90) and moderate for its subscales (0.73≤α≤0.87). The same study concluded good structure validity (four domains, Bi-factor model) and external validity (against a HA measure).

#### Short Health Anxiety Inventory (SHAI)

The Short Health Anxiety Inventory (SHAI) serves to quantify anxiety about health. It consists of 18 questions divided into two sections, 14 questions about the perceived likelihood of becoming ill (Main Section) and four questions corresponding to the perception of the negative consequences of an illness (Negative Consequences Section). Each question presents four options to be scored from 0 to 3, which allow obtaining a total score between 0 and 54 points (a score of 0 to 42 for the Main Section and 0 to 12 for the Section of Negative Consequences). The SHAI has validated versions in Spanish [30, 31], and for this study, the version adapted and validated in a South American country was used (Vallejo-Medina P, unpublished data, 2018). This study reported adequate construct validity and reliability (α = 0.82), useful for research purposes.

#### Statistical analysis

For the adaptation step (a), we calculated medians of the final outcomes from the expert opinion after finishing the Delphi method.

We started the psychometric validation (b) by summarising relevant information from each item. We calculated the average and standard deviation (SD) of each item, domain and the general CSS-12 scale. At the item level, we also calculated the inter-item correlation (polychoric correlation matrix), item-total correlation, item-rest correlation, and Cronbach’s alpha conditioned to the item elimination.

For assessing reliability, we estimated internal consistency and stability over time, as explained above. We considered Cronbach’s alpha and McDonald’s Omega values around 0.70 as adequate reliability, values around 0.80 as very good reliability, and values around 0.90 as excellent reliability [28, 34]. We calculated the Pearson’s r and the ICC for test-retest evaluation, overall and by domains. The Pearson’s r and ICC were interpreted as follows: 0,00–0,20 very poor reliability; 0,30–0,40 poor reliability; 0,50–0,60 moderate reliability; 0,70–0,80 high reliability; and >0,80 very high reliability [35].

For evaluating the structure validity, we compared four CSS-12 measurement models. These models were estimated by using a Weighted Least Square Mean and Variance (WLSMV) estimator and specified as follows:

1. Model-1: A one-dimensional model for which all items loaded in one general factor (no domains at all). This is a confirmatory factor analysis (CFA) model.
2. Model-2: A four-dimension model for which all factors (the four CSS-12 domains) are correlated. The model was specified as follows: EXC loads on items 1, 3, 6; COMP loads on items 2, 7, 10; DIST loads on items 4, 8, 9; and REAS loads on items 5, 11, 12. This is a confirmatory factor analysis (CFA) model.
3. Model-3: A model with four specific factors (EXC, COMP, DIST, REAS) and a general factor (CYB), all uncorrelated. The four specific factors were uniquely defined with their respective main loadings (i.e., factors linked to items as in Model 2), setting all other cross-loadings equal to zero. The general factor was defined through main loadings from all items. This is the Bifactor model evaluated by the CSS-12 creators [23].
4. Model-4: A model similar to Model 3, but instead of setting all other cross-loadings equal to zero, they are free parameters to estimate. This is a Bifactor-ESEM as described by Morin et al [36] and is our proposed model.

As visible, we built our modelling approach based on the original CSS-12 work by McElroy et al. [23]. In our approach, we preferred ESEM over EFA or CFA because ESEM 1) helps capture complex relationships among items and latent dimensions (not captured by EFA or CFA alone), providing a more accurate representation of the underlying structure, 2) by allowing cross-loadings and correlated uniquenesses, it can better account for the complexity and covariance structure in the data, resulting in improved model fit, 3) by allowing cross-loadings, it can help detect overextraction of factors or underextraction of relevant dimensions [32, 33, 37]. For the ESEM, we selected a Target rotation as we presumed a complex loading matrix structure, which means three or more factors and items with complexity 3 or more (items with three or more nonzero loadings) [32]. No specific constraints were imposed on the loadings, which allowed them to be freely estimated from the data and optimised during the analysis. More precisely, the factor loadings estimated in a standard EFA were used as the starting values for the corresponding factors in the ESEM. For ensuring reproducibility, the script of the four models is included in the supplemental material. The comparison of these models was based on their goodness of fit indexes. We included Tucker-Lewis Index (TLI) [38], Comparative Fit Index (CFI) [39], Root Mean Squared Error of Approximation (RMSEA) [40], and Standardized Root Mean Squared Residual (SRMR) [41]. Values of TLI and CFI ≥0.90 represent an acceptable model fit [42]. We consider RMSEA values ≤0.08 as acceptable and ≤0.05 as a very good model fit [43]. Values of SRMR between 0.05 and 0.08 are acceptable [41]. Additionally, we fitted models 1 to 4 using a Maximum Likelihood Estimation with Robust Standard Errors (MLR) estimator, allowing us to report the Akaike’s information criteria (AIC) and the sample-size adjusted Bayesian Information Criteria (BIC) for all models. We reported all the factor loadings for the Bifactor-ESEM model only. For assessing the uni-/multidimensionality of the Bifactor-ESEM model, we calculated two indices: (i) omega hierarchical (*ΩH*) and (ii) explained common variance (ECV) [44, 45]. *ΩH* reflects the proportion of the total variance attributable to the general CSS-12 factor (CYB) after controlling the effect for the specific factors EXC, COMP, DIST and REAS. Values >.80 imply that CYB is the main source of score variability [46]. ECV is the proportion of all the common variance that is expressed by the general CYB factor. ECV is ranged from 0 to 1, for which values >0.60 reflect uni-dimensionality [47, 48].

As explained previously, we evaluated the convergent model validity by calculating the correlation (Pearson’s r) between the SHAI and CSS-12 raw scales. Data management was supported by Microsoft Excel ® (Microsoft Corp, USA) and the statistical analysis by STATA® Version 16.0 (Stata Corp., College Station, Texas, USA) and Mplus™ Version 8.4 (Muthen and Muthen, USA).

#### Ethics

The Institutional Review Board of the Universidad Peruana de Ciencias Aplicadas reviewed and approved the study (approval number CEI / 211-10-18). All participants freely agreed to participate and gave their verbal informed consent after fully understanding the study objectives. All data were handled anonymously, in encrypted devices, and no risks were compromising sensitive information.

## Results

### (a) Adaptation to Spanish

The Delphi instrument evaluated by the five experts obtained scores ≥ 3 in each of the five aspects evaluated in the first round of review, with the acceptance of the instrument as culturally adapted. The final wording of the items is shown in the supplemental material.

### (b) Psychometric validation

#### CSS-12 scores

The CSS-12 total score average was 25.1 (SD 9.1). The average scores for each of the four dimensions were: Excessiveness = 7.8 (S.D. 2.8); Distress = 5.7 (S.D. 2.6); Reassurance = 5.8 (S.D. 2.6); and Compulsion = 5.8 (S.D. 2.5). Item mean ranged from 1.6 (SD 0.9) to 3.0 (SD 1.1), and item-total correlations ranged from 0.58 to 0.76. Item mean, standard deviation, item-total correlations and Cronbach’s alpha if removed are presented in **Table 1**. Polychoric correlation matrix between items of the CSS-12 is available in **S1 Table**.

**Table 1:** Means (M), standard deviations (SD), item-total correlations, item-rest correlations, and α if removed of CSS-12 items (N = 657).

*Item*	*M*	*SD*	*Item-total correlation*	*Item-rest correlation*	*Alpha if removed*
1	3.0	1.1	0.66	0.58	0.9231
2	2.4	1.1	0.76	0.70	0.9183
3	2.7	1.2	0.72	0.65	0.9204
4	2.0	1.0	0.76	0.70	0.9179
5	2.2	1.1	0.75	0.69	0.9185
6	2.1	1.0	0.81	0.76	0.9155
7	1.8	0.9	0.76	0.71	0.9178
8	1.8	1.0	0.73	0.67	0.9192
9	2.0	1.0	0.78	0.73	0.9169
10	1.6	0.9	0.74	0.69	0.9188
11	1.8	1.0	0.70	0.64	0.9203
12	1.9	1.0	0.75	0.70	0.9181

#### Reliability

The CSS-12 total score demonstrated excellent internal consistency (Cronbach’s alpha = 0.93; McDonald’s omega = 0.93), and internal consistency values were in the adequate–very good range for the subscales with Cronbach’s alpha (Cronbach’s alpha = 0.79–0.83; **Table 2**) and were very good with McDonald’s omega (Excessiveness: McDonald’s omega = 0.81; Distress: McDonald’s omega = 0.83; Reassurance: McDonald’s omega = 0.81; Compulsion: McDonald’s omega = 0.80).

**Table 2:** Reliability and convergent validity of the CSS-12.

Scales	Test retest Pearson’s r	Intraclass correlation	Cronbach’s alpha		SHAI
			Peruvian	English	Pearson’s r
**CSS-12** (CYB)	0,96	0,93	0,93	0, 90	0, 51
Excessiveness (EXC)	0,86	0,80	0,81	0, 83	0, 43
Distress (DIST)	0,89	0,89	0,83	0, 87	0, 51
Reassurance (REAS)	0,89	0,87	0,80	0, 73	0, 42
Compulsion (COMP)	0,84	0,83	0,79	0, 87	0, 41

SHAI, Short Health Anxiety Inventory

The Pearson’s correlation coefficients calculated from the test-retest for both the items and domains showed very high reliability, with all values being positive and >0.80 (**Tables 2 and 3**). The global intraclass correlation also revealed very high reliability for the instrument (ICC = 0.93) and domains (ICC = 0.80–0.89; **Table 2**).

**Table 3:** CSS-12 items and dimension to which they belong, mean and standard deviation (SD) of each item, and Pearson’s r for test-retest.

Dimension / Items	mean (SD)*	r Pearson**
**Excessiveness**		
p1. If I notice an unexplained bodily sensation I will search for it on the internet	2,96 (1,08)	0,74
p3. I read different web pages about the same perceived condition	2,73 (1,16)	0,72
p6. I enter the same symptoms into a web search on more than one occasion	2,09 (1,04)	0,79
**Distress**		
p4. I start to panic when I read online that a symptom I have is found in a rare/serious condition	1,96 (1,00)	0,79
p8. I think I am fine until I read about a serious condition online	1,77 (0,95)	0,62
p9. I feel more anxious or distressed after researching symptoms or perceived medical conditions online	1,98 (1,01)	0,80
**Reassurance**		
p5. Researching symptoms or perceived medical conditions online leads me to consult with my GP	2,18 (1,08)	0,85
p11. I suggest to my GP/medical professional that I may need a diagnostic procedure that I read about online (e.g. a biopsy/ a specific blood test)	1,76 (0,97)	0,89
p12. Researching symptoms or perceived medical conditions online leads me to consult with other medical specialists (e.g. consultants)	1,88 (1,01)	0,56
**Compulsion**		
p2. Researching symptoms or perceived medical conditions online distracts me from reading news/sports/entertainment articles online	2,35 (1,13)	0,65
p7. Researching symptoms or perceived medical conditions online interrupts my work (e.g. writing emails, working on word documents or spreadsheets)	1,78 (0,94)	0,56
p10. Researching symptoms or perceived medical conditions online interrupts my offline social activities (e.g. reduces time spent with friends/family)	1,63 (0,85)	0,82

CSS-12, Cyberchondria Severity Scale-12

(*) Means and Standard Deviations of CSS-12 Items (n = 657)

(**) Test-retest (n = 30)

#### Validity

Concerning construct validity, fit statistics for one-factor CFA, first-order four-factor CFA, Bifactor, and Bifactor-ESEM models of the CSS-12 are presented in **Table 4.** AIC and BIC values progressively improved from the most parsimonious one-factor model (Model 1) to the most complex Bifactor-ESEM model (Model 4). CFI, TLI, RMSEA and SRMR also improved their values in a similar way. This empirical evidence supports the most complex Bifactor-ESEM solution. The general CYB factor of the Bifactor-ESEM explains a considerable fraction of the shared variance (*ΩH* = 0.92; ECV = 0.79), allowing to sum the 12 items for obtaining a total score. Standardized factor loadings for this model are visible in **Table 5**.

**Table 4:** Goodness of fit of the four CSS-12 measurement models evaluated (N = 657).

Model	Short Description	CFI	TLI	RMSEA (90% CI)	SRMR	AIC*	BIC*
1	One-dimension (CFA)	0.943	0.930	0.141 (0.132–0.150)	0.052	18650.8	18698.0
2	Four-dimension (CFA)	0.959	0.944	0.126 (0.117–0.136)	0.043	18456.5	18644.9
3	Bifactor (original paper)	0.966	0.947	0.122 (0.112–0.133)	0.039	18189.5	18255.2
4	Bifactor-ESEM (our proposal)	0.999	0.997	0.029 (0.000–0.050)	0.006	18129.9	18227.0

(*) Models fitted with a Maximum Likelihood Estimation with Robust Standard Errors (MLR) estimator.

CFA, Confirmatory Factor Analysis; ESEM, exploratory structural equation modelling; AIC, Akaike’s information criteria; BIC, sample-size adjusted Bayesian Information Criteria; CFI, comparative fit index; TLI, Tucker–Lewis Index; RMSEA, Root Mean Square Error of Approximation; SRMR, standardised root mean square residual.

**Table 5:** Standardized factor loadings and internal consistency for the Bifactor-ESEM model (N = 657).

Item	CYB	EXC	COMP	DIST	REAS
1	**0.58** *	**0.53** *	0.03	0.06*	-0.04
3	**0.68** *	**0.52** *	0.07	-0.05	-0.08
6	**0.80** *	**0.24** *	0.06	0.08	-0.03
2	**0.72** *	0.31*	**0.37** *	-0.06	-0.01
7	**0.79** *	-0.02	**0.32** *	0.10*	0.02
10	**0.82** *	-0.21*	**0.22** *	0.17*	0.10
4	**0.74** *	0.17*	0.01	**0.39** *	-0.04
8	**0.78** *	-0.13*	0.08*	**0.33** *	0.08*
9	**0.79** *	0.01	0.04	**0.34** *	-0.03
5	**0.84** *	0.07	-0.19	-0.14	**-0.26**
11	**0.78** *	-0.08	-0.04	-0.02	**0.39**
12	**0.84**	-0.07	-0.03	-0.12	**0.22**
*α*	*0*.*93*	*0*.*81*	*0*.*83*	*0*.*80*	*0*.*79*

*p < 0.05

α, Cronbach’s alpha; CYB, Cyberchondria; EXC, excessiveness; COMP, compulsion; DIST, distress; REAS, reassurance.

Regarding convergent validity, the correlation between CSS-12 and SHAI was positive and moderate (Pearson’s r = 0.51), showing that some patients with health anxiety are likely to have cyberchondria as well. Weak to moderate correlation was evident when evaluating each subscale with SHAI (Pearson’s r = 0.41–0.51; **Table 2**).

## Discussion

The CSS-12 was successfully adapted to Spanish. It showed excellent reliability for the general CYB scale and good reliability for its subscales. The Bifactor-ESEM model with one general and four specific dimensions showed the best fit and strong one-dimensionality for the general CYB scale. The CSS-12 was positively correlated with the SHAI measure, confirming convergent validity against health anxiety. We are providing a formatted and free-to-use version of the Spanish CSS-12 in the supplemental material.

In general, the Spanish version of the CSS-12 has shown good psychometric properties like the original English version [23]. For example, general reliability is high for both the Peruvian and English populations (all college students), suggesting that CSS-12 internal consistency can be stable across populations. For the CSS-12 dimensions, Cronbach’s alpha remained adequate-to-good in the Spanish compared to the English version [23]. Likewise, the Spanish version is equally reliable than the full version of the CSS [16, 19]. For the Spanish version, we proposed and reported the McDonald’s omega, a reliability measure more accurate and with more feasible assumptions than Cronbach’s alpha [28, 49]. Although the latter is the most reported in previous CSS revisions, our findings showed that the true reliability could have been underestimated in those studies. Evidence from McDonald’s omega estimates and test-retest results of our study support the conclusion that all the CSS-12 dimensions have good reliability (>.80), including the Reassurance dimension (which reported just adequate reliability from Cronbach’s alpha).

A Bifactor solution for the CSS-12 has been largely justified in the literature. Bifactor models can capture common and specific variances separately, helping to understand the construct complexity more clearly [50]. For example, although REAS items are just moderately associated with this dimension, they are a strong representation of the general construct “cyberchondria” (CYB). This distinction cannot be recognised when other less complex models are fitted; for example, a first-order four-dimensional model. This advantage was early identified by the CSS-12 creators [23] and confirmed later by other independent researchers [51] providing support for using the general CYB score [52].

During our study, we explored the applicability of the Bifactor-ESEM model, a measurement model not previously evaluated for the CSS-12. This model represents a step forward towards the improvement of the cyberchondria measurement framework with the CSS. First, some studies conceptualized the full CSS as containing a general CYB factor orthogonal to its subfactors [20, 22]. Then, a more recent study developed the CSS-12, a shorter version of the original CSS, for which this Bifactor model was still the best measurement model for assessing cyberchondria [23]. Nevertheless, at this point, some items that can be substantively connected to more than one specific factor were assumed to be an expression of only one of them. In a Bifactor-ESEM model, there are no zero values pre-set; instead, these parameters are estimated under the assumption that some items load higher on one specific factor and lower on the others (but not necessarily zero). This allows the main estimates to be more accurate [32] and realistic in terms of what is happening with some items that express various facets of the cyberchondria simultaneously.

Prior research has revealed intriguing patterns within the cyberchondria. For example, certain EXC items have been observed to transition into the DIST and REAS dimensions [17]. Considering the conceptual framework of these subscales, the link to DIST can be elucidated by the notion that individuals with erroneous health beliefs may experience heightened distress when interpreting search results. This heightened distress might subsequently drive the inclination for excessive health-related information searches and the quest for reassurance through medical consultations. Similarly, the reclassification of an item from REAS to DIST may be attributed to the relationship between encountering worrisome information online and acting by visiting a healthcare provider [17]. In our study, the CSS-12’s item 2 was initially assigned to the COMP dimension only; however, it is substantively connected with item 1 of the EXC dimension, as our findings have demonstrated. We found that item 2 has a factor loading different from zero for EXC, which may be due to excessive time spent on online searches that interfere with other activities. A similar non-zero loading with more than one specific factor was confirmed for six of the twelve items.

Although the evidence shows the Bifactor model as one of the most promising, this cyberchondria measurement model is not perfect yet. In the Spanish CSS-12, the REAS dimension did not show a clear link with its items, conversely to the strong connection confirmed between the other items and their factors. Nevertheless, this problem is not new since the original English CSS-12 also showed a weak connection between item 5 and the REAS factor [23]. In the future, other cross-cultural revisions of the CSS-12 will enlighten this issue, helping to update the REAS factor measurement (e.g., readjusting one or more items). So far, the practical recommendation made by the CSS-12 authors -use and interpret results from the general CYB scale only–[23] is entirely valid and reliable for the Spanish version as well.

The Spanish and English versions of the CSS-12 have a similar convergent validity with the health anxiety (HA). Convergent validity between the English CSS-12 and the SHAI was previously reported [23], showing a positive correlation with a strength similar to the one we found for the Spanish version. Considering how easy it is to get access to health information online nowadays, it is likely that people with high levels of HA tend to use the Internet to obtain information about it [53] more frequently [54–56] and for longer [57]. This behaviour can trigger an increase in CYB level and sustain or increase the HA level itself [6]. During the creation of the CSS, authors have emphasized that, although CYB and HA are connected, they are not the same construct [7, 22, 23, 58]. For example, those with no prior HA may also experience stress because of searching about a health condition on the Internet [3]. For McElroy et al. [23], a critical factor that differentiates CYB from HA is EXC, which means that people spend an increasing amount of time searching about a health condition on the Internet, which affects their everyday lives [53]. This difference and the focus of the CSS-12 items on the Internet context justify its use as a CYB measure clearly differentiated from HA. Nevertheless, given that CYB is a relatively new construct, more studies are needed to further clarify this association and its directionality.

We have identified some limitations and strengths in our study. Given that the CSS-12 tool was only validated in students from a private Peruvian college, the external validity is limited. Although they are healthy in general, this population is commonly linked to “transient hypochondriasis”, which increases the levels of HA that usually occurs in medical students [59]. This could mark a distinction with the general population, depending on the academic year of study [60]. There were no previously validated tools to quantify CYB in the Spanish language, so this validation is the first to be carried out. This adapted and validated tool is short, practical and novel in the Latino and Spanish context, so its simple application will be valuable to expand knowledge regarding CYB. Although Peruvian Spanish is very similar to other Central and South American Countries’ Spanish (i.e., formal written Spanish, as in this CSS-12 version), some minor language differences could remain. As international guides state [61], it is at the user’s discretion to decide how appropriate this CSS-12 version is for evaluating local Spanish speakers.

In conclusion, the Spanish version of the 12-items Cyberchondria Severity Scale (CSS-12) was successfully adapted for the Peruvian college students’ population. It provides a valid and reliable unidimensional measure of the cyberchondria, which is distinguishable from the more general health anxiety. The Bifactor-ESEM model seems to offer a more accurate and realistic representation of the multifaceted nature of cyberchondria, contributing to a comprehensive understanding of this phenomenon. The CSS-12 can be applied for evaluating cyberchondria in similar populations and future research.

## Supporting information

S1 Fig Flowchart for participants selection. (TIF)

S1 Table Polychoric correlation matrix between items of the CSS-12. (PDF)

S1 File Mplus codes for statistical modelling. (PDF)

S2 File Spanish version: Cyberchondria Severity Scale—12 (CSS-12). (PDF)
