# Supplementary figures and images for: The short-form of the Cyberchondria Severity Scale (CSS-12): Adaptation and validation of the Spanish version in young Peruvian students
Source: PLoS One. 2023 Oct 5;18(10):e0292459. doi: 10.1371/journal.pone.0292459 (PMC10553310; doi:10.1371/journal.pone.0292459)

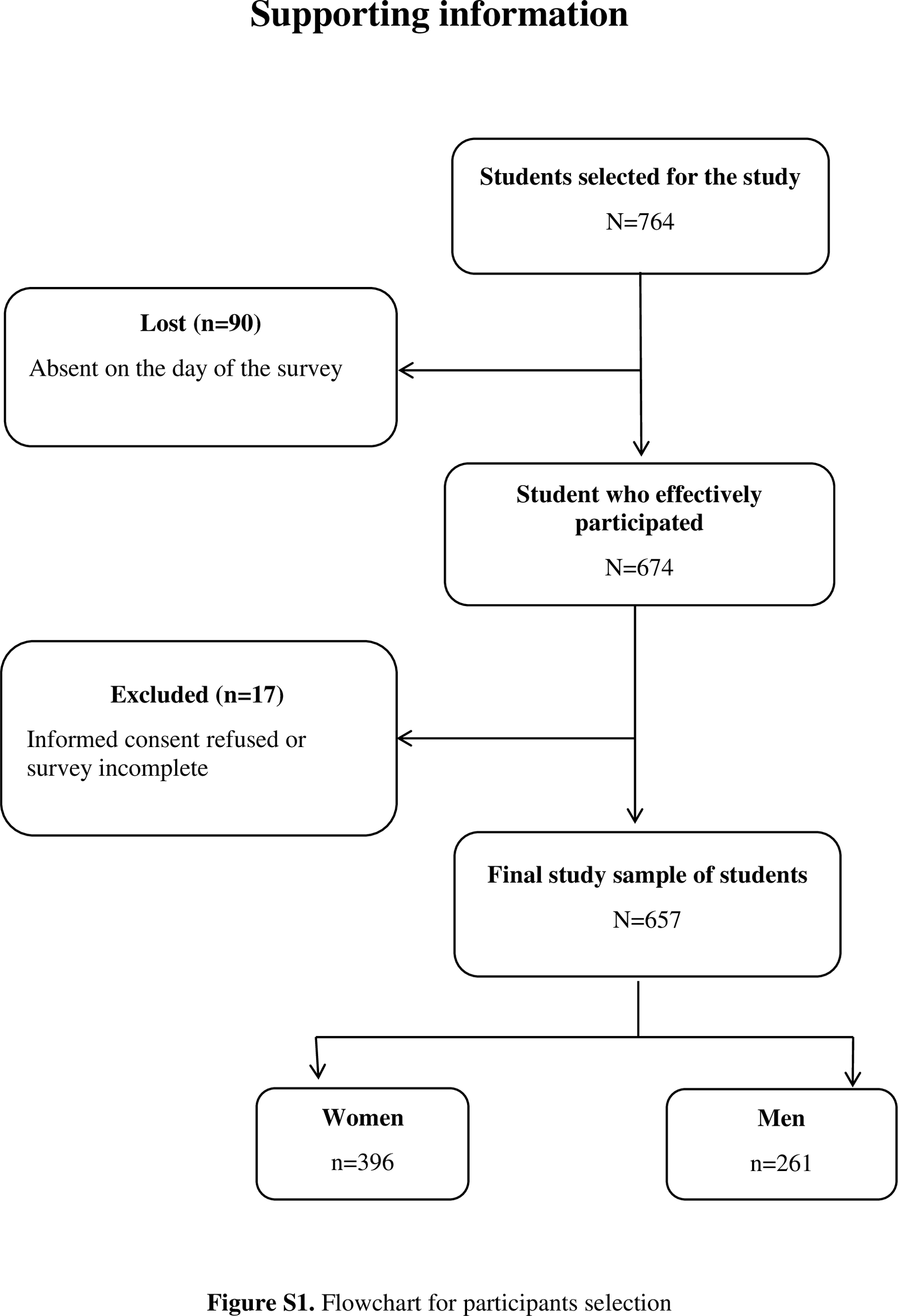

Supplement: S1 Fig — (TIF) [file pone.0292459.s001.tif]
