# Supplementary material for: The short-form of the Cyberchondria Severity Scale (CSS-12): Adaptation and validation of the Spanish version in young Peruvian students
Source: PLoS One. 2023 Oct 5;18(10):e0292459. doi: 10.1371/journal.pone.0292459 (PMC10553310; doi:10.1371/journal.pone.0292459)
Supplement: S1 Table — (PDF) [file pone.0292459.s002.pdf]

Supporting information

Table S1. Polychoric correlation matrix between items of the CSS-12

	CSS1	CSS2	CSS3	CSS4	CSS5	CSS6	CSS7	CSS8	CSS9	CSS10	CSS11	CSS12
**CSS1**	1											
**CSS2**	0,59	1										
**CSS3**	0,67	0,68	1									
**CSS4**	0,56	0,56	0,56	1								
**CSS5**	0,52	0,57	0,62	0,58	1							
**CSS6**	0,60	0,66	0,68	0,67	0,67	1						
**CSS7**	0,45	0,68	0,54	0,63	0,58	0,67	1					
**CSS8**	0,38	0,55	0,43	0,68	0,56	0,64	0,67	1				
**CSS9**	0,50	0,56	0,55	0,71	0,61	0,64	0,66	0,73	1			
**CSS10**	0,39	0,59	0,44	0,63	0,58	0,61	0,74	0,74	0,72	1		
**CSS11**	0,39	0,52	0,47	0,53	0,56	0,58	0,62	0,64	0,59	0,68	1	
**CSS12**	0,44	0,58	0,51	0,56	0,66	0,64	0,64	0,64	0,60	0,70	0,75	1
