# Supplementary material for: The short-form of the Cyberchondria Severity Scale (CSS-12): Adaptation and validation of the Spanish version in young Peruvian students
Source: PLoS One. 2023 Oct 5;18(10):e0292459. doi: 10.1371/journal.pone.0292459 (PMC10553310; doi:10.1371/journal.pone.0292459)
Supplement: S1 File — (PDF) [file pone.0292459.s003.pdf]

Supporting information

S1 File. Mplus codes for statistical modelling

Model 1

```
Title:
  CSS-12 (Model 1: One-dimension CFA)
Data:
  File is "[wherever it is]";
Variable:
  Names are
    id css1 css2 css3 css4 css5 css6 css7 css8 css9 css10 css11 css12;
  Usevariables are
    css1 css2 css3 css4 css5 css6 css7 css8 css9 css10 css11 css12;
  Categorical are all;
Analysis:
  estimator = wlsmv;
Model:
  css BY css1* css2 css3 css4 css5 css6 css7 css8 css9 css10 css11 css12;
  css@1;
Output:stdyx;
```

Model 2

```
Title:
  CSS-12 (Model 2: Four-dimension CFA)
Data:
  File is "[wherever it is]";
Variable:
  Names are
    id css1 css2 css3 css4 css5 css6 css7 css8 css9 css10 css11 css12;
  Usevariables are
    css1 css2 css3 css4 css5 css6 css7 css8 css9 css10 css11 css12;
  Categorical are all;
Analysis:
  estimator = wlsmv;
Model:
  excs BY css1* css3 css6;
  comp BY css2* css7 css10;
  dist BY css4* css8 css9;
  reas BY css5* css11 css12;
  excs@1 comp@1 dist@1 reas@1;
Output:stdyx;
```

Model 3

```
Title:
  CSS-12 (Model 3: Bifactor <- McElroy et al)
Data:
  File is "[wherever it is]";
Variable:
  Names are
    id css1 css2 css3 css4 css5 css6 css7 css8 css9 css10 css11 css12;
  Usevariables are
    css1 css2 css3 css4 css5 css6 css7 css8 css9 css10 css11 css12;
```

```
  Categorical are all;
Analysis:
  parameter = theta;
  estimator = wlsmv;
Model:
  css BY css1* css2 css3 css4 css5 css6 css7 css8 css9 css10 css11 css12;
  css@1;
  excs BY css1* css3 css6;
  excs@1;
  comp BY css2* css7 css10;
  comp@1;
  dist BY css4* css8 css9;
  dist@1;
  reas BY css5* css11 css12;
  reas@1;
  css WITH excs@0 comp@0 dist@0 reas@0;
  excs WITH comp@0 dist@0 reas@0;
  comp WITH dist@0 reas@0;
  dist WITH reas@0;
Output:stdyx;
```

Model 4

```
Title:
  CSS-12 (Model 4: Bifactor-ESEM)
Data:
  File is "[wherever it is]";
Variable:
  Names are
    id css1 css2 css3 css4 css5 css6 css7 css8 css9 css10 css11 css12;
  Usevariables are
    css1 css2 css3 css4 css5 css6 css7 css8 css9 css10 css11 css12;
  Categorical are all;
Analysis:
  estimator = wlsmv;
  rotation = target (orthogonal)
Model:
  css BY css1 css2 css3 css4 css5 css6 css7 css8 css9 css10 css11 css12 (*1);
  excs BY css1 css3 css6
      css2~0 css7~0 css10~0 css4~0 css8~0 css9~0 css5~0 css11~0 css12~0 (*1);
  comp BY css2 css7 css10
      css1~0 css3~0 css6~0 css4~0 css8~0 css9~0 css5~0 css11~0 css12~0 (*1);
  dist BY css4 css8 css9
      css1~0 css3~0 css6~0 css2~0 css7~0 css10~0 css5~0 css11~0 css12~0 (*1);
  reas BY css5 css11 css12
      css1~0 css3~0 css6~0 css2~0 css7~0 css10~0 css4~0 css8~0 css9~0 (*1);
Output:stdyx;
```
