# Supplementary material for: The short-form of the Cyberchondria Severity Scale (CSS-12): Adaptation and validation of the Spanish version in young Peruvian students
Source: PLoS One. 2023 Oct 5;18(10):e0292459. doi: 10.1371/journal.pone.0292459 (PMC10553310; doi:10.1371/journal.pone.0292459)
Supplement: S2 File — (PDF) [file pone.0292459.s004.pdf]

Supporting information

S2 File. Spanish Version: Cyberchondria Severity Scale - 12 (CSS-12)

Instrucciones para los autores

Se emplea una escala de Likert, del 1 al 5, los puntajes van de 3 a 15 por dimensión y de 12 a 60 para la escala global.

Escala	*Descripción*	*Ítems*
Excesividad	Naturaleza creciente o repetida de las búsquedas	1, 3, 6
Angustia	Ansiedad o angustia como resultado de las búsquedas	4, 8, 9
Seguridad	Búsquedas que conducen a los individuos a buscar un consejo médico profesional	5, 11, 12
Compulsión	Búsquedas web que interfieren con otros aspectos de la vida dentro y fuera del internet	2, 7, 10

Escala de Severidad de la Cibercondría - 12 (CSS-12)

Lea las siguientes afirmaciones e indique cómo se aplican normalmente a usted. Tenga en cuenta que este cuestionario se refiere a las condiciones médicas percibidas; es decir, las condiciones aparentes que cree que podría tener y no a las condiciones que han sido diagnosticadas por una profesión médica. Cabe resaltar que cuando hacemos referencia a búsquedas en línea nos referimos a las efectuadas en Internet.

	Nunca	Casi nunca	A veces	Con frecuencia	Siempre
1) Si noto una sensación corporal extraña, la buscaré en Internet	1	2	3	4	5
2) Investigar en línea los síntomas o las condiciones médicas percibidas me distrae de leer artículos de noticias/deportes/entretenimiento en línea	1	2	3	4	5
3) Leo diferentes páginas web sobre la misma condición médica percibida	1	2	3	4	5
4) Entro en pánico cuando leo en línea que un síntoma que tengo se encuentra asociado a una condición rara / grave	1	2	3	4	5
5) Investigar síntomas o condiciones médicas percibidas en línea me lleva a consultar con mi médico de cabecera	1	2	3	4	5
6) Ingreso los mismos síntomas en una búsqueda web en más de una ocasión	1	2	3	4	5
7) Investigar en línea síntomas o condiciones médicas percibidas interrumpe mi trabajo (por ejemplo, escribir correos electrónicos, trabajar en documentos de Word u hojas de cálculo)	1	2	3	4	5
8) Creo que estoy bien hasta que leo sobre una condición grave en línea	1	2	3	4	5
9) Me siento más ansioso o angustiado después de investigar en línea sobre síntomas o condiciones médicas percibidas	1	2	3	4	5
10) Investigar en línea sobre síntomas o posibles condiciones médicas percibidas interrumpe mis actividades sociales fuera de línea (reduce el tiempo que paso con amigos / familiares)	1	2	3	4	5
11) Le sugiero a mi médico que puedo necesitar un procedimiento de diagnóstico sobre el que haya leído en línea (por ejemplo, una biopsia / un análisis de sangre específico)	1	2	3	4	5
12) Investigar en línea sobre síntomas o condiciones médicas percibidas me lleva a consultar con otros especialistas médicos además de mi médico	1	2	3	4	5
